# Mean amplitude deviation calculated from raw acceleration data: a novel method for classifying the intensity of adolescents’ physical activity irrespective of accelerometer brand

**DOI:** 10.1186/s13102-015-0010-0

**Published:** 2015-08-07

**Authors:** Minna Aittasalo, Henri Vähä-Ypyä, Tommi Vasankari, Pauliina Husu, Anne-Mari Jussila, Harri Sievänen

**Affiliations:** 1 The UKK Institute for Health Promotion Research, PL 30, 33501 Tampere, Finland; 2 National Institute for Health and Welfare, PL 30, 00271 Helsinki, Finland

**Keywords:** Adolescents, Physical activity, Accelerometer, Raw acceleration data, Classification

## Abstract

**Background:**

Using raw acceleration data to assess the intensity of physical activity enables direct comparisons between studies using different accelerometer brands. Mean amplitude deviation (MAD in mg) calculated from resultant tri-axial raw acceleration signal was recently shown to perform best in classifying the intensity of physical activity in adults irrespective of the accelerometer brand. This study compared MAD values and cut-points between two different accelerometers in adolescents.

**Methods:**

Twenty voluntary participants (10 girls and 10 boys) of average age of 14 wore two accelerometers (Actigraph GTX3, Pensacola FL, USA and Hookie AM13, Espoo, Finland) and heart rate monitors (M61, Polar Electro Oy, Kempele, Finland) while completing ten 2-min patterns of typical activities ranging from sedentary behaviour to light, moderate and vigorous-intensity locomotion. Bland-Altman method examined the agreement of MAD values between the accelerometers. Correlation coefficient between individual heart rates and MAD values indicated the validity of pattern-based intensity classification. Generalized ordinal logistic regression determined the intensity-specific MAD cut-points for both accelerometers.

**Results:**

MAD values varied from 3 mg (lying supine) to 1609 mg (running). Hookie gave higher values than Actigraph in accelerations exceeding 700 mg. The correlation coefficient between MAD values and heart rates was 0.96 for Hookie and 0.97 for Actigraph. Respectively, the MAD cut-points were 29 and 27 (light), 338 and 330 (moderate), and 604 and 558 (vigorous).

**Conclusions:**

MAD values and cut-points of Hookie and Actigraph showed excellent agreement. Analysing raw accelerometer data with MAD values may enable the comparison of accelerometer results between different studies also in adolescents.

## Background

Physical activity has been proven highly beneficial for health throughout the lifespan [1]. The interest in assessing physical activity and sedentary behaviour in children and adolescents has received increasing attention as obesity and related health risks have become more general also at younger age [2, 3] and the evidence about the health benefits of physical activity in paediatric population has strengthened [4, 5].

Until recent years the knowledge and thus the recommendations on young people’s physical activity have relied mainly on data obtained from surveys. However, the subjectivity and recollection bias inherent in surveys have long been acknowledged in assessing the wide spectrum of health-enhancing physical activity in adults [6] and sporadic physical activity typical for youth [7–9]. As a result, assessment methods considered more objective, such as accelerometers, have become common also in large-scale studies of children and adolescents.

Numerous accelerometer brands and models are nowadays available for the objective assessment of physical activity. Generally, they are considered suitable for assessing the intensity of movements and to produce information on the quantity of overall physical activity [10]. Majority of accelerometers convert the raw acceleration signal to counts per minute to get a generic measure of the intensity of physical activity. The counts are then classified into different intensity levels by using cut-points. However, the algorithms behind the counts vary by accelerometer brand and model [11] and studies use different cut-points in classifying the intensity [12] both compromising the direct comparison of studies. In youth, the count-based cut-points have also been shown to misclassify the intensity of physical activity between 33 and 68 % of the time [13]. As a result, the use of raw acceleration data expressed in direct physical acceleration units such as G-forces rather than in calculated activity counts has been recommended for analysing the intensity of physical activity [9, 14]. A universal method is consequently needed for analysing the raw acceleration signal by intensity in order to produce comparable information across the accelerometer brands and studies.

It was recently shown in adults that the mean amplitude deviation (MAD) of raw acceleration signal performed best irrespective of accelerometer brand in separating sedentary and pace-specific ambulatory activities from each other [15]. Also the cut-points based on MAD values were equal between the accelerometer brands in classifying the intensity of physical activity. The applicability of this promising method need to be examined also in youth, which may produce different accelerations in similar activities when compared to adults. This study compares MAD values and MAD cut-points between two different accelerometer brands (Actigraph GTX3, Actigraph LLC., Pensacola FL, USA, http://www.actigraphcorp.com and Hookie AM13, Traxmeet Ltd, Espoo, Finland, http://company.traxmeet.com) in adolescents. If the MAD values and cut-points were found similar with both accelerometers, MAD would offer a universal method also in youth to classify the intensity of PA derived from raw acceleration data regardless of the accelerometer brand used.

## Methods

### Participants

Twenty voluntary adolescents aged 13 to 15 years were recruited to the study via personal contacts. The participants and one of their guardians signed a written informed consent to participate in the study, which was conducted according to the ethical principles of The Finnish National Advisory Board on Research Ethics (http://www.tenk.fi/en/ethical-review-human-sciences) and was approved by the University of Tampere and Tampere Area Ethical Review Board (http://www.uta.fi/english/research/ethics/review/committee.html).

### Procedures

After reporting their height and weight the participants one by one completed a supervised array of ten 2-min physical activities illustrating typical free-living patterns. The activities were assigned to the intensity levels based on their pattern: *sedentary behaviour* (lying supine on a bed, sitting on a chair, sitting while working on a computer, standing and standing while moving 1 kg-handweights on a table surface), *light activity* (slow walking, normal walking), *moderate activity* (brisk walking) and *vigorous activity* (jogging, running). The array of activities was the exactly same as was recently used in adults [15].

A heart rate monitor was used to confirm the validity of pattern-based classification of intensity. The chest strap of the heart rate monitor (M61, Polar Electro Oy, Kempele, Finland, http://www.polarelectro.fi) was fastened around the participant’s chest and the wrist unit was attached to the participant’s right wrist. The researcher recorded the heart rate from the wrist unit in the beginning of each 2-min activity, one minute after initiating the activity and in the end of the activity.

The ambulatory tasks were performed on a 100-m inside track and the paces relative to verbal instructions (slow, normal, brisk, jogging, running) were self-selected by the participants. The researcher observed the participants during the activities and timed the 2-min interval of each activity with a stopwatch. Fifteen-second break was allocated for changing from one activity to another.

During the activities the triaxial Hookie and Actigraph accelerometers were worn on the same elastic belt. Both devices were fixed firmly to a standard position: Hookie to the right and Actigraph to the left hip. The Hookie AM13 device employs a 100Hz sampling frequency and a ±16 000 mg (milligravity units) tri-axial dynamic range and the Actigraph GT3X device 30Hz and ±3000 mg, respectively.

### Data processing

The data from both accelerometers was collected in raw mode, which provides the acceleration data in actual G-forces. The sampled triaxial acceleration data were stored onto a workstation and analysed in epoch lengths corresponding approximately 5 s, which has been shown to have the lowest root mean squared error in assessing children’s physical activity with Actigraph [16] and to be sufficient for assessing MAD in the steady type of activities not involving rapid spurts [15]. Thus, the number of data points included in the analysed epoch was 512 for Hookie (epoch length 5.1 s) and 128 for Actigraph (epoch length 4.3 s).

MAD describes the mean distance of data points about the mean ($MAD=\frac{1}{n}\ {\displaystyle \sum}\left|{r}_i - \overline{r}\right|$), where *n* is the number of samples in the epoch, *r*_*i*_ the i^th^ resultant sample within the epoch and $\overline{r}$ the mean resultant value of the epoch. From the measured raw acceleration data, the MAD values (mg) of the resultant acceleration within three epochs (5 s after the beginning of given activity, in the middle, and 5 s before the end of given activity) were calculated for both accelerometers. The more detailed description about the calculation of resultant acceleration and MAD can be found from Vähä-Ypyä *et al.* [15].

### Statistics

Mean and standard deviation (SD) of MAD values are given as descriptive statistics. Bland-Altman method was used to examine the agreement of MAD values between the accelerometers. In doing so, the difference in the MAD values between the accelerometers in 10 different activities was plotted against their mean during the same activities. An example of the averaged minute-by-minute MAD values across all activities including the time changing from one activity to another was also performed in relation to single participant’s accelerations to illustrate the comparability of MAD values in more real-world conditions as suggested by Welk *et al.* [17].

To confirm the validity of pattern-based classification of intensity, within-subject correlation coefficients were calculated between the heart rates and MAD values. Here, the activities representing sedentary behaviour were excluded from the analysis because in some subjects - most likely due to the emotional excitement related to the measurements in the beginning of the session - the heart rate was higher during sedentary behaviour than during light activity. Pearson correlation coefficients between the mean heart rate at 1 and 2 min during each activity and the MAD values were calculated for each subject separately. Then, a mean correlation coefficient was determined by performing z-transformation of subject-specific coefficients taking the mean of these values and the inverse z-transformation of the mean.

MAD cut-points (mg) for classifying the activity patterns by intensity were defined for both accelerometers with generalized ordinal logistic regression by using the compound symmetry structure, which assumes that the correlations between the repeated measures are uniform. Misclassification represented the proportion (%) of MAD values showing incorrect classification. In addition, misclassification was also assessed using the respective universal cut-points for intensity classification in adults [15].

## Results

Twenty adolescents – 10 girls and 10 boys - volunteered and participated in the study (Table 1). Their mean age was 14.2 years (SD 0.7), height 168.0 cm (9.7), weight 56.8 kg (10.9) and body mass index 20.0 kg/m^2^ (2.3).

**Table 1:** Basic characteristics of the participants: means, standard deviations (SD) and ranges

	Girls (n = 10)		Boys (n = 10)		All (n = 20)	
	Mean (SD)	Range	Mean (SD)	Range	Mean (SD)	Range
Age, years	14.3 (0.7)	13 to 15	14.0 (0.8)	13 to 15	14.2 (0.7)	13 to 15
Height, cm	164.4 (9.4)	148 to 180	171.5 (9.0)	158 to 183	168.0 (9.7)	148 to 183
Weight, kg	54.5 (10.4)	40 to 70	59.1 (11.4)	46 to 87	56.8 (10.9)	40 to 87
Body mass index, kg/m^2^	20.0 (2.2)	16.0 to 22.8	20.0 (2.5)	16.8 to 26.0	20.0 (2.3)	16 to 26

**Fig. 1:**
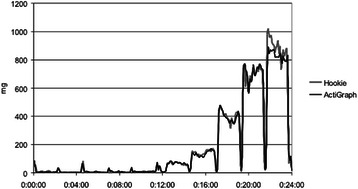
An example of the minute-by-minute comparison of mean absolute deviation (MAD, mg) calculated from the Hookie and Actigraph acceleration data of a single participant across all 10 activities

Table 2 shows the MAD values for each subject in each activity broken down by gender. Hookie gave somewhat higher accelerations in both jogging and running compared with Actigraph and a similar but smaller difference was observed in brisk walking (Fig. 1).

**Table 2:** Actigraph and Hookie accelerations (mean absolute deviation, mg) in the 10 activities by gender. The lower part of the table presents the cut-points from sedentary behaviour to light activity (Cut 1), from light to moderate activity (Cut 2) and from moderate to vigorous activity (Cut 3) and their specificity, sensitivity and accuracy in classifying the intensity of activities

**Fig. 2:**
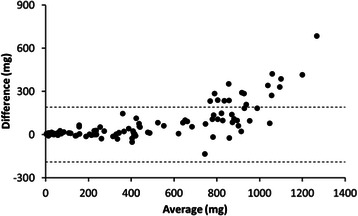
Difference in the mean absolute deviation (MAD, mg) calculated from Hookie and Actigraph acceleration data in all 10 activities plotted against the mean of MAD values in the same activities. Dashed lines show 95 % limits of agreement

According to Bland Altman analysis the accelerometers agreed well in measuring MAD values up to 700 mg (Fig. 2). After that the level of difference between the accelerations increased systematically Hookie giving higher values.

**Fig. 3:**
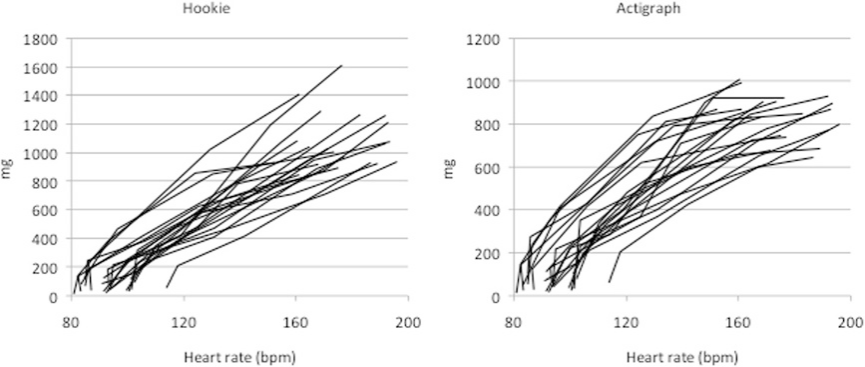
Individual mean absolute deviations (MAD, mg) calculated from Hookie (left panel) and Actigraph (right panel) acceleration data plotted against the mean heart rate within five activities from slow walking to running

The mean correlation coefficient between MAD values and heart rates across activities from slow walking to running was 0.96 for Hookie and 0.97 for Actigraph indicating strong linearity between heart rate and acceleration supporting thus the validity of pattern-based intensity classification (Fig. 3). The mean heart rate was 92 (SD 8) in slow walking, 99 (12) in normal walking, 118 (15) in brisk walking, 155 (19) in jogging and 180 (13) in running.

Compared to Actigraph, Hookie showed similar cut-points for separating sedentary behaviour from slow walking (29 and 27 mg), brisk walking from normal walking (338 and 330 mg) and somewhat higher for jogging from running (604 and 558 mg). Misclassification rate was low for both accelerometers (2.5 % or lower). Hookie misclassified altogether 7 and Actigraph 9 activities out of 200. Using the universal cut-points (17, 331 and 599) derived from adult accelerometer data in similar setting [15], the misclassification rate was again low (3 % or lower). With these cut-points Hookie misclassified 9 and Actigraph 8 activities.

## Discussion

A need for using raw acceleration data instead of activity counts for measuring the intensity of physical activity has been expressed widely [18–20]. This study compared the MAD-based cut-points of two different accelerometer brands, a widely used Actigraph GTX3 and a new Hookie device, in classifying the intensity of basic free-living physical activities in adolescents. To our knowledge this is the first study to utilize a raw acceleration data based trait (*i.e.,* MAD) in comparing two accelerometer brands among adolescents.

According to the results both accelerometers provided virtually equal MAD values up to about 700 mg and showed almost perfect agreement in classifying activities into light, moderate and vigorous categories with marginal misclassification rate. Our findings indicate the utility of MAD cut-points in the intensity-based classification of adolescents’ raw acceleration data. Noteworthy, the cut-points observed in the present data of adolescents were similar to those obtained in adults [15], and indeed, the cut-points performed equally well in adults and adolescents. In sum, it may be possible to use same cut-points for classifying locomotion by intensity both in adolescents and adults.

The study raised some concerns, which should receive attention in the future. First, both accelerometers sampled the acceleration signal at sufficient 30 and 100Hz rates for detecting general movements comprising frequencies typically less than 10Hz [14]. However, the wider dynamic range of Hookie (±16,000 mg) outperformed Actigraph (±3000 mg) by covering also the accelerations during vigorous activities such as jogging and running. Also, the higher 100Hz sampling frequency of Hookie accounted at least to some extent for the higher MAD values and thus the cut-points in moderate and vigorous intensity physical activity compared with Actigraph. By using the new Actigraph GT3X+, which can employ 100 Hz sampling frequency and has the dynamic range of ±8000 mg, these differences may disappear and the agreement of the cut-points might have been almost perfect.

Second, the sample size of the present study was too small preventing us from reliable comparison of MAD cut-points between girls and boys although Table 2 indicates that boys may have slightly higher cut-point from sedentary to light activity than girls. The limited sample size hindered us also to stratify the subjects according to common anthropometric and biomechanical variables, such as body mass or height, to evaluate the consistency of the results in various subgroups. For example, the recent treadmill study by Horner *et al.* [21] involving young, active females indicate that soft tissue (subcutaneous adiposity) under the sensor may explain individual differences and variability in hip-derived accelerometer output in walking. Thus far, the applicability of the findings in various subgroups is left open. However, given the fact that the measurement is based on a direct physical trait *i.e.,* movement induced acceleration and that the universal MAD cut-points, similar to those found in adolescents in the present study, have been proven suitable for classifying the intensity of physical activity in adults [15], it is highly likely that the present findings will apply to other groups of adolescents as well.

Third, it is acknowledged that the array of 10 different activities was quite narrow covering only a fraction of the most usual weight-bearing activities and leaving out a wide spectrum of activities, where the activity-induced accelerations may not be accurately determined such as swimming, cycling, skiing, climbing, gym exercising etc. Moreover, the activities were performed under supervision and in exceptional surroundings, which was likely to lead to some deviation from habitual performance. The correspondence to real life may also have been impaired by the short duration of single activity, which was only 2 min instead of 4 to 5 min suggested by Welk *et al.* [17]. However, if the activity was performed similarly over the time period of interest the acceleration values would have remained unchanged irrespective of the duration of the activity. Using longer duration would have prolonged the length of the whole protocol up to an hour possibly slowing down the recruitment process and hampering the concentration of teenagers, who may be more impatient than adults in performing rigorous tasks.

Fourth, not measuring the distance walked or run prevented us from standardizing the paces and defining the intensity of ambulatory tasks more objectively. We were, therefore, unable to verify how well our pattern-based intensity classification corresponded to pace-specific intensity levels. Heart rate, which was used in this study to assess the validity of pattern-based classification of intensity, is known to be susceptible for psychological reactions [9]. As more elevated heart rates were observed in sedentary behaviour than in light activity, activities classified to sedentary behaviour had to be excluded from testing the correlation between acceleration levels and heart rates. In terms of light, moderate and vigorous activities the within-individual correlations between the MAD and the heart rate were very high for both accelerometers suggesting a valid physiological association between the MAD value and intensity as judged from the incident heart rate.

Finally, it can be argued that the placement of the monitors to the different sides of the hip may have influenced on MAD values and data interpretation. However, in a previous study by McClain *et al.* [22], which was conducted in free-living conditions, the intraclass correlations for various accelerometer outputs (activity counts, steps, intensity-specific minutes) between the right and left hip placement were high ranging from 0.97 to 0.99. Thus, it is unlikely that the accelerometer placement had notable impact on MAD values and their interpretation in the present study.

## Conclusions

The MAD cut-points of the two different accelerometer brands with different technical specifications were almost identical and indicate that it is possible to find a method, which classifies similarly the intensity of adolescents’ physical activity from raw acceleration data irrespective of accelerometer brand. This is promising because using a universal trait (*i.e.,* MAD) in the analysis enables us to compare and synthesize results from studies using different accelerometers and eventually to make more reliable conclusions, for example, about the predictors and health impacts of intensity-specific physical activity. However, the finding should be confirmed with larger sample size and age-range as well as wider spectrum of free-living activities and accelerometer brands.

## Abbreviation

MAD: Mean amplitude deviation

## References

[1] Physical Activity Guidelines Advisory Committee. Physical Activity Guidelines Advisory Committee Report. U.S. Department of Health and Human Services, Washington, DC; 2008. http://www.health.gov/paguidelines/guidelines/.

[2] WHO. The challenge of obesity - quick statistics. http://www.euro.who.int/en/health-topics/noncommunicable-diseases/obesity/data-and-statistics.

[3] De Onis M, Blössner M, Borghi E (2010). Global prevalence and trends of overweight and obesity among preschool children. Am J Clin Nutr.

[4] Strong WB, Malina RM, Cameron JR, Blimkie CJR, Daniels SR, Dishman RK (2005). Evidence based physical activity for school-age youth. J Pediatr.

[5] Janssen I, LeBlanc AG (2010). Systematic review of the health benefits of physical activity and fitness in school-aged children and youth. Int J Behav Nutr Phys Act.

[6] Prince SA, Adamo KB, Hamel ME, Hardt J, Connor-Gorber S, Tremblay M (2008). A comparison of direct versus self-report measures for assessing physical activity in adults: a systematic review. Int J Behav Nutr Phys Act.

[7] Baquet G, Stratton G, Van Praagh E, Berthoin S (2007). Improving physical activity assessment in prepubertal children with high-frequency accelerometry monitoring: a methodological issue. Prev Med.

[8] Adamo KB, Prince SA, Tricco AC, Connor-Gorber S, Tremblay M (2009). A comparison of indirect versus direct measures for assessing physical activity in the pediatric population: a systematic review. Int J Pediatr Obes.

[9] Corder K, Ekelund U, Steele RM, Wareham NJ, Brage S (2008). Assessment of physical activity in youth. J Appl Physiol.

[10] Staudenmayer J, Zhu W, Catellier DJ (2012). Statistical considerations in the analysis of accelerometry-based activity monitor data. Med Sci Sports Exerc.

[11] Trost SG, O’Neil M (2014). Clinical use of objective measures of physical activity. Br J Sports Med.

[12] Crouter SE, DellaValle DM, Haas JD, Frogillo EA, Bassett DR (2013). Validity of Actigraph 2-regression model, Matthews Cut-points, and NHANES cut-points for assessing free-living physical activity. J Phys Act Health.

[13] Trost SG, Loprinzi PD, Moore R, Pfeiffer KA (2011). Comparison of accelerometer cut-points for predicting activity intensity in youth. Med Sci Sports Exerc.

[14] Chen KY, Janz KF, Zhu W, Brychta RJ (2012). Redefining the roles of sensors in objective physical activity monitoring. Med Sci Sports Exerc.

[15] Vähä-Ypyä H, Vasankari T, Husu P, Suni J, Sievänen H (2014). A universal, accurate intensity-based classification of different physical activities using raw data of accelerometer. Clin Physiol Funct Imaging.

[16] McClain JJ, Abraham TI, Brusseau TA (2008). Tudor-Locke C. Epoch length and accelerometer outputs in children: Comparison to direct observation. Med Sci Sports Exerc.

[17] Welk GJ, McClain J, Ainsworth BE (2012). Protocols for evaluating equivalency of accelerometry-based activity monitors. Med Sci Sports Exerc.

[18] John D, Freedson P (2012). Actigraph and Actical physical activity monitors: a peek under the hood. Med Sci Sports Exerc.

[19] Cain KL, Sallis JF, Conway TL, Van Dyck D, Calhoon L (2013). Using accelerometers in youth physical activity studies: a review of methods. J Phys Act Health.

[20] Troiano RP, McClain JJ, Brychta RJ, Chen KY (2014). Evolution of accelerometer methods for physical activity research. Br J Sports Med.

[21] Horner FE, Slade J, Bilzon JLJ (2013). The effect of anatomical placement and trunk adiposity in the reliability and validity of triaxial accelerometer output during treadmill exercise. J Phys Act Health.

[22] McClain JJ, Sisson SB, Tudor-Locke C (2007). Actigraph accelerometer interinstrument reliability during free-living in adults. Med Sci Sports Exerc.

